# AMPAR interacting protein CPT1C enhances surface expression of GluA1-containing receptors

**DOI:** 10.3389/fncel.2014.00469

**Published:** 2015-02-02

**Authors:** Esther Gratacòs-Batlle, Natalia Yefimenko, Helena Cascos-García, David Soto

**Affiliations:** ^1^Laboratori de Neurobiologia, Area de Neurobiologia Cellular i Molecular, Institut d'Investigació Biomèdica de Bellvitge-IDIBELLL'Hospitalet de Llobregat, Spain; ^2^Department of Pathology and Experimental Therapeutics, Faculty of Medicine, University of BarcelonaL'Hospitalet de Llobregat, Spain

**Keywords:** glutamate receptors, GluA1, CPT1C, AMPAR trafficking, surface expression, electrophysiological recordings, cortical neurons, palmitoylation

## Abstract

AMPARs mediate the vast majority of fast excitatory synaptic transmission in the brain and their biophysical and trafficking properties depend on their subunit composition and on several posttranscriptional and posttranslational modifications. Additionally, in the brain AMPARs associate with auxiliary subunits, which modify the properties of the receptors. Despite the abundance of AMPAR partners, recent proteomic studies have revealed even more interacting proteins that could potentially be involved in AMPAR regulation. Amongst these, carnitine palmitoyltransferase 1C (CPT1C) has been demonstrated to form an integral part of native AMPAR complexes in brain tissue extracts. Thus, we aimed to investigate whether CPT1C might be able to modulate AMPAR function. Firstly, we confirmed that CPT1C is an interacting protein of AMPARs in heterologous expression systems. Secondly, CPT1C enhanced whole-cell currents of GluA1 homomeric and GluA1/GluA2 heteromeric receptors. However, CPT1C does not alter the biophysical properties of AMPARs and co-localization experiments revealed that AMPARs and CPT1C are not associated at the plasma membrane despite a strong level of co-localization at the intracellular level. We established that increased surface GluA1 receptor number was responsible for the enhanced AMPAR mediated currents in the presence of CPT1C. Additionally, we revealed that the palmitoylable residue C585 of GluA1 is important in the enhancement of AMPAR trafficking to the cell surface by CPT1C. Nevertheless, despite its potential as a depalmitoylating enzyme, CPT1C does not affect the palmitoylation state of GluA1. To sum up, this work suggests that CPT1C plays a role as a novel regulator of AMPAR surface expression in neurons. Fine modulation of AMPAR membrane trafficking is fundamental in normal synaptic activity and in plasticity processes and CPT1C is therefore a putative candidate to regulate neuronal AMPAR physiology.

## Introduction

Glutamate is the neurotransmitter involved in the majority of excitatory synaptic processes in the brain. This amino acid activates primarily ionotropic glutamate receptors (iGluRs): NMDA, AMPA, and Kainate receptors. Amongst iGluRs, the AMPA receptors (AMPARs) are essential as they mediate 90% of the fast excitatory neurotransmission in the central nervous system (CNS). Although their main role relates to synaptic transmission, AMPARs are also responsible for some forms of activity-dependent synaptic plasticity, the process thought to underlie higher order cognitive functions such as learning and memory (Barry and Ziff, 2002; Malinow and Malenka, 2002; Song and Huganir, 2002; Bredt and Nicoll, 2003).

AMPARs are tetrameric structures formed by four different subunits: GluA1–A4 and can be found as homo- or heterotetrameric structures (Traynelis et al., 2010), heteromeric receptors being the most common combinations found in neurons, amongst different brain regions (Gallo et al., 1992; Kondo et al., 1997; Lu et al., 2009; Reimers et al., 2011). Their subunit composition is crucial for AMPAR properties and their roles in neurons largely rely on the different intracellular carboxyl-terminal (C-terminal) domains, which vary between subunits. AMPAR subunits can be found with long (GluA1, GluA2-long, and GluA4) or short (GluA2, GluA3, and GluA4-short) intracellular C-terminal domains (Köhler et al., 1994). The different C-termini of AMPAR subunits permit a great variability in protein-to-protein interactions dependant on both the distinct AMPAR subunits (Palmer et al., 2005) and the class of PDZ binding domain (Sheng and Sala, 2001; Cai et al., 2002). The C-terminal domain of GluA subunits also contains most of the well-characterized phosphorylation and palmitoylation sites. These posttranslational modifications allow a fine and complex regulation of AMPARs through the specific interaction of the receptor with multiple intracellular proteins, which play crucial roles in AMPAR trafficking and function (Anggono and Huganir, 2012; Lu and Roche, 2012).

Of the multiple proteins that transiently interact with AMPARs and that determine their trafficking, synaptic targeting and recycling in neurons, some special attention must be given to transmembrane proteins that form integral part of the functional receptor. In addition to trafficking, these proteins modulate channel gating properties hence acting as genuine auxiliary subunits of the AMPARs. Amongst these, the most important are the *Transmembrane AMPA receptor Regulatory Proteins* (TARPs; Kato et al., 2010a; Straub and Tomita, 2012). Indeed, the vast majority of AMPARs in the CNS are associated with TARPs (Menuz et al., 2008; but see Schwenk et al., 2009) and they appear to be crucial for correct trafficking and synaptic targeting (Tomita et al., 2005). Depending on the TARP subtype, AMPAR trafficking properties are differentially modulated resulting in a differential synaptic integration (Jackson and Nicoll, 2011).

A recent proteomic study confirming the interaction of AMPARs with transient and integral partners of AMPARs has also identified a number of proteins capable of interacting with AMPAR subunits. One of them is Carnitine palmitoyltransferase 1C (CPT1C), which forms part of some macromolecular complexes of AMPARs in the brain (Schwenk et al., 2012). This protein is a member of Carnitine palmitoyltransferases, a family of enzymes that catalyzes the exchange of acyl groups between carnitine and CoA to facilitate the transport of long chain fatty acids from the cytoplasm to the mitochondria for β-oxidation (McGarry and Brown, 1997). CPT1C is a specific CPT1 brain isoform strongly expressed in the hypothalamus, the hippocampus, cortex, and cerebellum (Price et al., 2002). CPT1C is highly homologous to the other CPT1s: it has the ability to bind palmitoyl-CoA and maintains the same binding affinity as CPT1A for malonyl-CoA (the endogenous allosteric CPT1 inhibitor). However, CPT1C has a 100-fold lower catalytic activity than the other isoforms (Sierra et al., 2008). Moreover, it is located in the endoplasmic reticulum (ER) instead of the mitochondria (Sierra et al., 2008; Carrasco et al., 2012). The molecular mechanism of CPT1C action has not been unraveled yet, but some clues about its importance in mammalian brain function derive from CPT1C knockout mice studies. These KO mice show an impairment of motor functions, muscle strength, hypoactivity (Carrasco et al., 2013), behavioral learning deficits (Carrasco et al., 2012) and altered maturation of dendritic spines in hippocampal neurons indicating an important role of CPT1C in the CNS. Additionally, some results indicate that CPT1C is also involved in the control of food intake and energy expenditure (Wolfgang et al., 2006). It has also been described that a gain-of-function of CPT1C in the brain of transgenic mice results in severe growth retardation and in a reduction of brain weight (Reamy and Wolfgang, 2011).

In the present study we investigate whether CPT1C might affect AMPAR function. Our results confirm that GluA subunits are able to interact with CPT1C and this interaction modulates AMPAR surface expression in a subunit-dependent manner, without altering the gating properties of the receptor. Moreover we find that the palmitoylable cysteine residue located in the 585 position of GluA1 is crucial for CPT1C modulation of AMPAR surface level. Since it is clear that regulation of AMPAR membrane trafficking is critical for normal synaptic activity and for several forms of synaptic plasticity in the brain, the involvement of CPT1C in these processes is relevant for understanding AMPAR physiology.

## Materials and methods

### Expression constructs

AMPAR subunit cDNAs were a gift from Prof. Dr. Stephen Heinemann (Salk Institute, La Jolla, CA) and Prof. Dr. Peter Seeburg (Max Planck Institute, Heidelberg, Germany). pDs-Red-ER-KDEL was a generous gift of Juan Pablo Muñoz (IRB, Barcelona). GluA1-pIRES-mCherry: a pIRES vector expressing GluA1 and mCherry translated from a single bicistronic mRNA (used for heteromeric GluA1/GluA2 electrophysiological experiments). CPT1C plasmid vectors were a generous gift from Dr. Núria Casals (Universitat Internacional de Catalunya) and CPT1A-GFP was a gift from Dr. Dolors Serra (Universitat de Barcelona). Characteristics of CPT1C and CPT1A plasmid vectors were: (1) CPT1C-GFP: a plasmid containing CPT1C cDNA sequence C-terminally tagged with EGFP; this construct produces a protein of approximately 100 kDa (all experiments involving CPT1C have been performed with this plasmid unless otherwise stated); (2) CPT1C-pIRES: a pIRES vector expressing CPT1C and EGFP translated from a single bicistronic mRNA and (3) CPT1A-GFP: contains CPT1A cDNA sequence C-terminally tagged with EGFP. All cDNAs are from rat and plasmid vectors are all under the control of the same promoter (CMV promoter).

To obtain GluA1 cDNAs with mutations in the palmitoylation sites we used site-directed mutagenesis to change specific base pairs. Primers containing the mutation/s were designed and then synthetized by Integrated DNA Technologies (IDT). GluA1(C585S) and GluA1(C811S) mutant cDNAs resulted from changing the codon TGT to TCT and TGC to TCC, respectively. Both changes produce a cysteine to serine switch making these palmitoylation targets disappear. For the double mutant GluA1(C585,811S) we used GluA1(C585S) cDNA as a template and introduced the C811S mutation to create the GluA1(C585,811S) product, in which both palmitoylation sites from GluA1 were eliminated. The primers used for introducing the mutations were the following:

**C585S:** AAGGAT**C**TGACATTTCCCCCAGGTCCC
**C811S**: CCTTAATCGAGTTCT**C**CTACAAATCCCGTAGCG

All constructs were fully sequenced to verify sequence integrity.

### Cell culture and transfection

tsA201 cells – derived from HEK293, which do not express CPT1C protein (Sierra et al., 2008)—were maintained in DMEM:F12 containing 10% fetal bovine serum and 1% penicillin–streptomycin solution in 5% CO_2_/95% air at 37°C. 24 h before transfection, 1.5 × 10^6^ cells were plated into T25 flasks for coimmunoprecipitation (Co-IP) and Acyl Biotin Exchange assays (ABE) or 0.5 × 10^5^ cells onto poly-D-lysine-coated coverslips for immunofluorescence (IF) and electrophysiological (EP) experiments. Cells were transiently co-transfected with 5.4 μg total cDNA (for Co-IP and ABE) and 0.6 μg total cDNA (for IF and EP) using X-tremeGENE transfection reagent (Roche) according to the manufacturers' directions. In all transfections the ratio used was 1:2 (GluA:CPT1C). Media was replaced 24 h after transfection with fresh media containing 2,3-dioxo-6-nitro-1,2,3,4-tetrahydrobenzo[f]quinoxaline-7-sulfonamide 50 μM (NBQX; Tocris-ABCam, Abcam) to prevent AMPAR-mediated toxicity. For EP experiments, cells were re-plated on glass coverslips to allow optimal density. All experiments were performed 48 h later.

### Neuronal cultures and transfection

Cortical neuron cultures were prepared from mouse embryos (E18). The cerebral cortex was isolated and maintained in cold Hank's Balanced Salt Solution (HBSS, Gibco) supplemented with 0.45 % glucose. After removal of the meninges, the cortical tissue was digested mildly with trypsin for 17 min at 37°C and mechanically dissociated. Cells were washed three times in HBSS and resuspended in Neurobasal medium supplemented with 2 mM Glutamax (Gibco) before filtering in 70 μm mesh filters (BD Falcon). Cells were plated onto glass coverslips (5 × 10^4^ cells/cm^2^) coated with 0.1 mg/ml poly-L-lysine (Sigma). 2 h after seeding, the plating medium was replaced by complete growth medium (Neurobasal medium supplemented with 2% B27 (Invitrogen) and 2 mM Glutamax) and the coverslips were incubated at 37°C in a humidified 5 % CO_2_ atmosphere. Every 3–4 days, half of the conditioned medium was removed and replaced by fresh growth medium. Primary cultures were transfected with Lipofectamine 2000 on day 7 *in vitro* (7 DIV), according to the manufacturer's, instructions and the cells were fixed 72 h after transfection. All the experimental procedures were carried out according to European Union guidelines (Directive 2010/63/EU) and following protocols that were approved by the Ethics Committee of the Bellvitge Biomedical Research Institute (IDIBELL).

### Coimmunoprecipitation

48 h after transfection, tsA201 cells were washed twice with room temperature PBS and collected in 1 ml 50 mM Tris-HCl (pH 7.4) with Protease Inhibitor cocktail (Sigma) on ice. All subsequent steps were performed at 4°C. Cells were lysed in a Polytron (VDI 12; VWR) at force 5, for 20 s, twice. Lysates were centrifuged at 1000 ×g for 10 min to pellet nuclei and unlysed cells. The supernatant was further centrifuged at 20,000 ×g for 30 min, and the membrane fraction (pellet) was resuspended in solubilisation buffer (1 % Triton X-100, 150 mM NaCl and 50 mM Tris-HCl pH 8, containing protease inhibitors) and homogenized with a Polytron for 20 s. After 20 min on ice, insoluble material was pelleted with a 30 min centrifugation at 20,000 ×g and the supernatant was quantified using the BCA method (Thermo Scientific). 200–400 μg of total protein were incubated with 4 μg of antibody overnight at 4°C with orbital agitation (antibodies: mouse anti-GluA1-NT (N-terminus), rabbit anti-GluA2 (cytoplasmic domain) both from Merck Millipore, rabbit serum anti-GFP from Invitrogen). Antibody-protein complexes were pulled down by incubating with 80 μl of Protein-A sepharose beads (Sigma) pre-equilibrated with solubilisation buffer, for 2 h. Precipitated complexes were washed in solubilisation buffer three times and eluted with 2 × SDS/DTT sample buffer, heated 10 min at 76°C and separated on SDS/PAGE. Before adding the antibodies, 10% of total protein (100 μl) was removed as input samples. 500 μl of pre-cooled acetone was added to the input samples, the mixture was vortexed and incubated overnight at −20°C. The precipitated proteins were cleared by centrifugation at 20,000 ×g for 20 min, supernatant was removed, and pellets were air-dried for 15–30 min and resuspended with appropriate volume of 2 × SDS/DTT buffer.

### Immunoblotting

Samples were separated by SDS/PAGE in 4-15% mini-protean TGX precast gels, transferred using Trans-Blot Turbo transfer system on nitrocellulose membranes (all from BioRad). Membranes were blocked in TBS with 0.1% Tween 20 (TBS-T) containing 5% (wt/vol) BSA or non-fat skim milk. Peroxidase-conjugated sheep anti-mouse or donkey anti-rabbit secondary antibodies (Dako) diluted in blocking solution at 1:1000 dilution, were detected by using ECL reagent (Amersham) and a LAS3000 Intelligent Dark Box (Fujifilm) was used to report western-blots. Quantification of the western blots was performed with Image J (NIH).

### Immunocytochemistry

Immunofluorescence was performed in tsA201 cells grown on lysine treated coverslips, 48 h after transfections. Washes were always performed by immersion of the coverslips in PBS or PBS-G (20 mM Glycine in PBS). Composition of different solutions: Fixation solution (2% PFA in PBS), permeabilization solution (0.1% Triton X-100 in PBS-G), blocking solution (10% NGS, 2% BSA, 0.1% Triton X-100 in PBS-G), antibody incubation solution (4% normal goat serum and 0.1% BSA in PBS-G) and triton-antibody solution (antibody incubation solution containing 0.1% Triton X-100). Antibodies incubations were performed in a humid chamber at 37°C for 1 h.

For co-localization of CPT1C with GluA1 or GluA2 and for determining the level of surface expression of GluA1 in tsA201, the following method was used: staining surface AMPARs was achieved by labeling live cells with the mouse anti-GluA1-NT or mouse anti-GluA2 antibodies (both from Merck Millipore) in a 1:200 solution in DMEM:F12, for 7–10 min at 37°C. In neuronal cultures, the surface staining step was performed for 1 h at 37°C on fixed neurons (4% PFA + 4% sucrose). After washes in room temperature PBS, tsA201 cells were fixed for 15 min at room temperature and incubated with goat anti-mouse Alexafluor 555 (Molecular Probes) at 1:250 in antibody incubation solution. After several washes in PBS-G, cells were fixed again to preserve the binding of the first secondary antibody. Cells were subsequently permeabilized for 5–10 min and blocked for 30 min. Next, and in order to determine the total expression of AMPARs in each cell, GluA1 or GluA2 were labeled incubating the coverslips with the same mouse anti-GluA1-NT or mouse anti-GluA2 antibodies at 1:200 (in triton-antibody incubation solution). Following washes in PBS-G, cells were incubated with goat anti-mouse Alexafluor 647 (Molecular Probes) at 1:500 (in triton-antibody incubation solution). Coverslips were then washed and mounted with Fluoromount (Invitrogen).

For co-localization of CPT1C with GM-130 (Golgi marker), cells were transfected with CPT1C-GFP and Golgi staining was performed on fixed, permeabilized and blocked tsA201 cells by incubating cells with mouse anti-GM-130 (BD-Biosciences) at 1:50 in triton-antibody incubation solution and subsequently with goat anti-mouse Alexafluor 555 (Molecular Probes) at 1:500.

For co-localization of CPT1C with an ER marker we co-transfected tsA201 cells with 600 ng of total DNA at a ratio of 1:2 (ER-KDEL: CPT1C). 48h after transfection cells were fixed for 15 min in 4% PFA, washed and mounted in Fluoromount.

### Confocal imaging and immunofluorescence quantification

Confocal images were acquired on a spectral confocal microscope (Leica TCS-SL, CCiTUB), using 40× or 63× oil objective lenses, in multitracking mode to minimize channel crosstalk. Each image was taken through laser excitation lines 488, 543, and 633 and Differential Interference Contrast (DIC). For immunofluorescence quantification experiments the same settings for each condition and throughout experiments were used. For tsA201 cells, stacks of 0.7 μm were taken from different areas and for cortical neurons stacks were of 0.3 μm.

Quantification of GluA1 surface expression was performed using Image J (NIH). Each stack was Z-projected to the maximum intensity. With the freehand selection tool individual cells or dendrites co-expressing the receptor and CPT1C-GFP or GFP (expression verified by tracking fluorescence intensity in the green channel) were traced and fluorescence in each channel was measured. The fluorescence values of each cell/dendrite were then analyzed, red integrated density (INTDEN) being the value corresponding to surface expression of the receptor in that cell/dendrite, and blue INTDEN being the value for total expression of GluA1 in the same cell/dendrite (tsA201 cells with low levels of blue fluorescence were not quantified). The mean background intensity was obtained from three different areas of each image and subtracted from each measurement using the following formula:

(1) $$\text{CTCF}=\text{INTDEN}-({\text{AREA}}^{*}\text{ MEAN FLUORESC BKGD})$$

where CTCF stands for corrected total cell fluorescence. Then, the ratio surface to total was obtained from the CTCF value from red fluorescence (surface) divided by the CTCF value from blue fluorescence (total), and normalized to the reference condition (GluA1+GFP or GFP transfected neurons). Finally, column graphics including the mean ratio of each condition were plotted and the error bars (SEM) were obtained. A set of at least 3 different immunofluorescences for each condition was performed and 10–50 cells of each condition were analyzed for each immunofluorescence. For neuronal experiments, three separate independent cultures were performed and 70 and 80 dendrites from 21 and 23 neurons from control and test condition were analyzed.

Quantification of co-localization was performed using the Manders Overlap Coefficient (MOC) calculated in Image J via the JACoP plugin from images of single cells. This coefficient ranges between 1 and zero with values close to 1 being high co-localization, and values close to zero being low.

### Electrophysiology: general procedures

Cells were visualized with an inverted microscope (IX50; Olympus). Electrodes were fabricated from borosilicate glass (1.5 mm o.d., 0.86 mm i.d., Harvard Apparatus) pulled with a PC-10 vertical puller (Narishige). Electrode resistance varied between configurations (see below). Macroscopic currents were recorded at room temperature (22-25°C) in the whole-cell configuration (wc) or from outside-out patches (o) excised from GFP-positive cells. Currents were recorded with Axopatch 200B amplifier, filtered at 2 kHz (wc) or 10 kHz (o) and digitized at 5 kHz (wc) or 50 kHz (o) using Digidata 1440A interface with pClamp 10 software (Molecular Devices Corporation). For all configurations the “extracellular” solution contained (in mM): 145 NaCl, 2.5 KCl, 1 CaCl_2_, 1 MgCl_2_, 10 glucose and 10 HEPES (pH to 7.42 with NaOH). For fast agonist application, 10 mM glutamate was added to the “extracellular” solution. The “intracellular” solution contained (in mM): 145 CsCl, 2.5 NaCl, 1 Cs-EGTA, 4 MgATP, and 10 HEPES (pH to 7.2 with CsOH). Spermine tetrahydrochloride (Sigma Aldrich) was added to intracellular solution at 100 μM in all cases.

### Whole-cell recordings

Whole-cell recordings were made from isolated cells using thick-walled electrodes with a resistance of 3–5 MΩ, giving a final series resistance of 5–15 MΩ. A voltage ramp protocol was used to change the holding potential (0 to –80 mV then to +80 mV at a rate of 160 mV/s; with the voltage held at –80 mV for 200 ms previous to the ramp). Receptors were activated by a bath application of 1 mM glutamate plus 25 μM cyclothiazide (CTZ) to prevent receptor desensitization. Control traces were subtracted from stable agonist-activated responses and the average current recorded at −80 mV was measured. In all recordings, to control for differences in cell surface area, the response to glutamate was expressed as current density (–pA/pF; maximum current divided by input capacitance as measured from the amplifier settings).

The rectification index (RI) was defined as the absolute value of glutamate-evoked current at +60 mV divided by that at −60 mV:

(2) $$\text{RI}+60\text{mV}/-60\text{mV}=\left|{\text{I}}_{+60\text{mV}}\right|\text{/}\left|{\text{I}}_{-60\text{mV}}\right|$$

### Agonist application to excised patches

For out-side out patches we used electrodes with a final resistance of 8–12 MΩ. Rapid agonist application was achieved by switching between a continuously flowing control solution (extracellular solution diluted by 4%) and a glutamate-containing solution (extracellular solution plus 2.5 μg/ml sucrose and 10 mM glutamate). Solution switching was achieved by piezoelectric translation of a theta-barrel application tool made from borosilicate glass (1.5 mm o.d.; Sutter Instruments) mounted on a piezoelectric translator (P-601.30; Physik Instrumente). 100 ms jumps were applied to outside-out patches at a holding potential of −60 mV. At the end of each experiment, the adequacy of the solution exchange was tested by destroying the patch and measuring the liquid-junction current at the open pipette (10–90% rise time normally 200–300 μs).

The kinetics of desensitization of the glutamate-activated currents were determined by fitting the glutamate-evoked responses at *V*_m_ −60 mV to a double-exponential function in order to determine the weighted time constant (τ_w,des_):

(3) $$\tau_{w,\text{des}}=\tau_{\text{f}}\left(\frac{{\text{A}}_{\text{f}}}{{\text{A}}_{\text{f}}+{\text{A}}_{\text{s}}}\right)+\tau_{\text{s}}\left(\frac{{\text{A}}_{\text{s}}}{{\text{A}}_{\text{f}}+{\text{A}}_{\text{s}}}\right)$$

where A_f_ and τ_f_ are the amplitude and time constant of the fast component of desensitization and A_s_ and τ_s_ are the amplitude and time constant of the slow component of desensitization.

### Non-stationary fluctuation analysis (NSFA)

To deduce channel properties from macroscopic responses, glutamate (10 mM) was applied to outside-out patches (100 ms duration, 1 Hz, *V*_hold_ −60 mV) and the ensemble variance of all successive pairs of current responses were calculated. The single channel current (i) and the total number of channels in the patch (N) were calculated by plotting this ensemble variance against mean current (I) and fitting with a parabolic function:

(4) $$\sigma^{2}=\sigma_{\text{B}}^{2}+\left(\text{i}\bar{\text{I}}-\left(\frac{{\bar{\text{I}}}^{2}}{\text{N}}\right)\right)$$

where σ^2^_B_ is the background variance. Along with normal peak-to-peak variation in the currents due to stochastic channel gating, some patches presented a gradual decline in peak amplitude. The mean response was calculated from the periods of the recordings showing stable responses that were identified using a Spearman rank-order correlation test (Igor Pro with Neuromatic). The weighted-mean single-channel conductance was determined from the single-channel current and the holding potential corrected for the calculated liquid-junction potential (+4.9 mV; pClamp 10).

### Acyl-biotin exchange (ABE) assay

Detection of palmitoylation levels of GluA1 subunits was performed as described in Brigidi and Bamji (2013). 48 h after transfection, tsA201 cells co-expressing GluA1 and GFP or GluA1 and CPT1C-GFP, were washed with ice-cold PBS and lysed with a 30 G syringe 6 times in ice-cold lysis buffer (1% IGEPAL, 50 mM Tris-HCl pH 7.5, 150 mM NaCl, 10% Glycerol, Protease Inhibitor Cocktail (Roche) and PMSF) containing 50 mM N-ethylmaleimide (NEM) (Sigma). All steps where performed at 4°C. Lysates were cleared by centrifugation at 16,000 xg for 30 min and the amount of protein in the supernatant was determined using the BCA method (Thermo Scientific). 750 μg – 1.5 mg of protein were used for overnight immunoprecipitation of GluA1 [4 μg of anti-GluA1-NT antibody (Merck Millipore)]. Then, protein-antibody complexes were pulled-down with Protein-A sepharose beads (Sigma) for 1–2 h. The total immunoprecipitate was then resuspended in lysis buffer with 10 mM NEM and it was split into two equivalent samples: one sample for specific cleavage and unmasking of the palmitoylated cysteine's thiol group by 1 M hydroxylamine treatment (+HAM sample) and a second equivalent sample but without the presence of HAM to control non-specific incorporation of biotin (-HAM sample). Before performing HAM treatment, samples were totally washed to avoid any presence of unbound NEM. 1 M HAM solution was prepared in pH 7.2 lysis buffer and ± HAM treatment was performed for 1 h at room temperature. After washes, selective labeling of the palmitoylated cysteine using a thiol-reactive biotinylation reagent, biotin-BMCC (1 μM) (Thermo Scientific) in pH 6.2 lysis buffer was performed for 1 h at 4°C in ± HAM samples. Then, the thiol-biotinylated proteins following the ABE steps were resolved by SDS-PAGE and Western Blotting was performed. Membranes were blocked with 3% BSA in TBS-T and incubated with Streptavidin-HRP (Invitrogen) (1:5000 from a 1 mg/ml stock in 0.3 % BSA). After stripping, the same membrane was incubated with an anti-GluA1-NT antibody (1:1000) to normalize palmitoylation levels to the amount of immunoprecipitated protein.

### Analysis and statistics

Electrophysiological recordings were analyzed using IGOR Pro (Wavemetrics Inc.) with NeuroMatic (Jason Rothman, UCL). Data are presented in the text as the mean ± SEM from n patches and in the figures as bar plots of the group mean, with error bars denoting the SEM. Comparisons between two groups were performed using the non-parametric Mann-Whitney U test. Differences were considered significant at *p* < 0.05. Statistical analysis was performed using GraphPad Prism version 5.0d for Mac OS X (GraphPad Software, San Diego California USA, www.graphpad.com).

## Results

### AMPAR subunits coimmunoprecipitate with CPT1C in heterologous expression system

**Figure 1:**
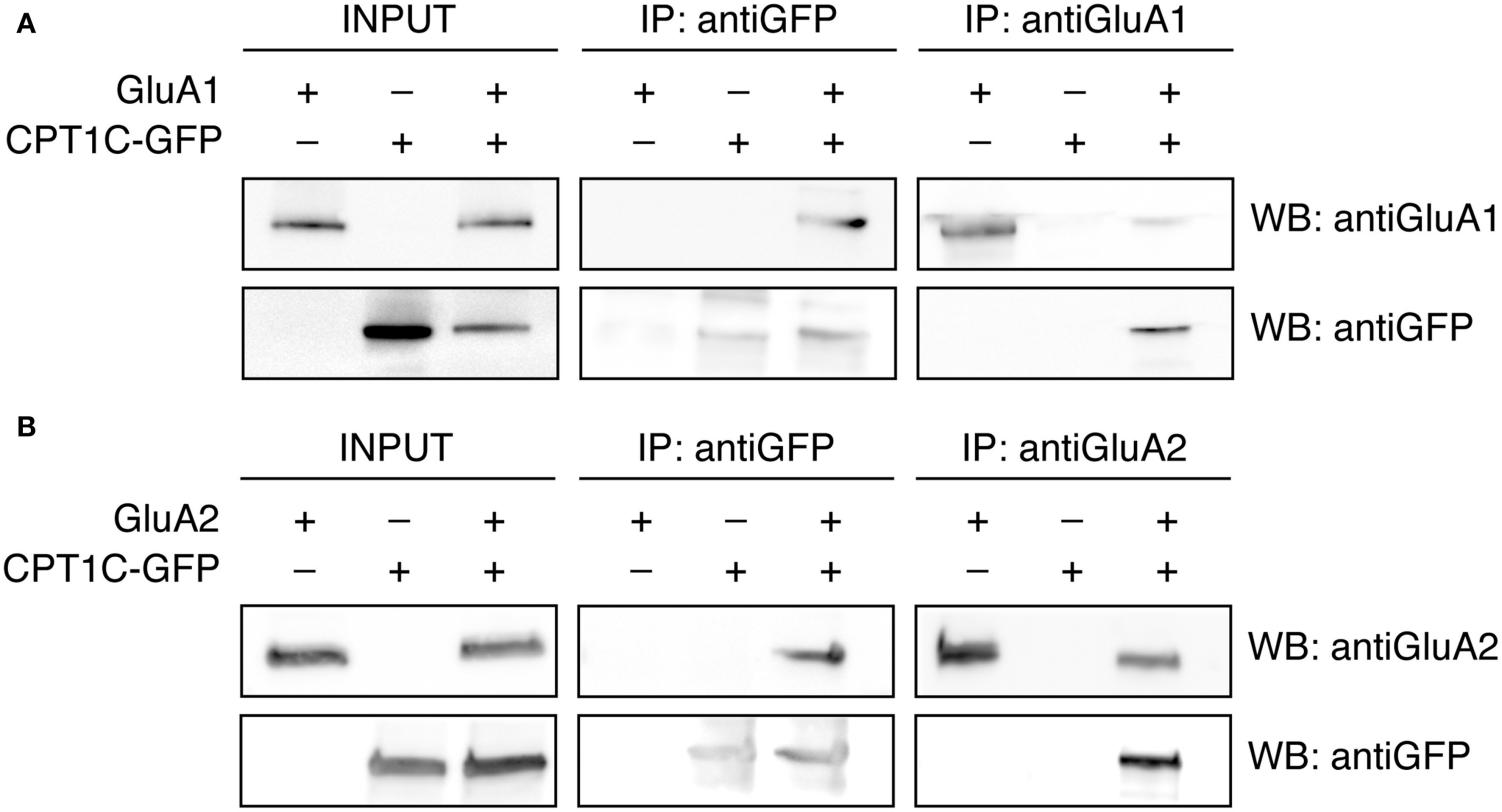
**GluA1 and GluA2 coimmunoprecipitate with CPT1C in expression systems. (A)** Co-IP of the membranous fraction of tsA201 cells co-expressing either GluA1 alone or together with CPT1C-GFP confirming the interaction between CPT1C and GluA1. As a negative control GluA1 was co-expressed with an empty plasmid expressing GFP alone (pEGFP) and CPT1C-GFP was co-expressed with an empty plasmid (pcDNA3.0). Transfected cells were lysed and membranes were solubilized. 200-400 μg of solubilized membranes was immunoprecipitated with an anti-GFP antibody (IP: antiGFP) or with anti-GluA1-NT antibody (IP: antiGluA1). An input sample collected prior to immunoprecipitation of these extracts is shown as “INPUT.” Inputs and immunoprecipitated samples were separated using SDS-PAGE and Western Blot was performed using anti-GluA1-NT (WB: antiGluA1) or anti-GFP (WB: antiGFP) antibodies. Immunoprecipitations were performed three times. **(B)** Same as in **(A)** but for tsA201 cells expressing GluA2 or GluA2 plus CPT1C-GFP. Membrane lysates were immunoprecipitated with an anti-GFP antibody (IP: antiGFP) or a rabbit polyclonal anti-GluA2 (cytoplasmic domain) (IP: antiGluA2). Western Blots were performed using mouse anti-GluA2 (WB: antiGluA2) or anti-GFP (WB: antiGFP) antibodies. Immunoprecipitations were replicated three times.

CPT1C has been shown to interact with native AMPAR subunits in rat brain tissue (Schwenk et al., 2012). Thus we first sought to determine if in our expression system CPT1C also interacted with AMPAR subunits. To analyse the interaction of GluA1 with CPT1C we performed coimmunoprecipitation assays from tsA201 cells transiently transfected with GluA1 and the construct CPT1C tagged with the green fluorescence protein GFP (CPT1C-GFP; see methods) (Figure 1). Membranes of these cells were immunoprecipitated either with anti-GluA1 or anti-GFP and subsequently detected using Western blot. Antibodies recognizing GFP were able to pull down GluA1 when co-expressed with CPT1C-GFP (Figure 1A; upper middle panel). The ~100 KDa band was not detected with GluA1 antibody in anti-GFP immunoprecipitates from cells transfected only with GluA1 or CPT1C-GFP. Additionally, coimmunoprecipitation was observed by pulling down CPT1C-GFP with an anti-GluA1 antibody (Figure 1A; lower right panel). Correct expression of the different constructs was confirmed by Western blot of the input samples (Figure 1A; upper and lower left panels). Our results confirm previous observations and indicate that in tsA201 cells CPT1C physically associates with GluA1 subunit.

Equivalent conditions were used for detecting interaction of GluA2 with CPT1C and the same negative controls were performed. Figure 1B shows that GluA2 subunit coimmunoprecipitated with CPT1C-GFP when the latter was pulled-down with an anti-GFP antibody (upper middle panel). Additionally, the anti-GluA2 antibody was able to pull-down CPT1C (Figure 1B; lower right panel). The presence of both proteins was detected in the input samples (Figure 1B; upper and lower left panels). These results confirm that both GluA1 and GluA2 interact with CPT1C in tsA201 cells when both proteins are co-expressed.

### CPT1C increases whole-cell currents of GluA1-containing AMPARs

**Figure 2:**
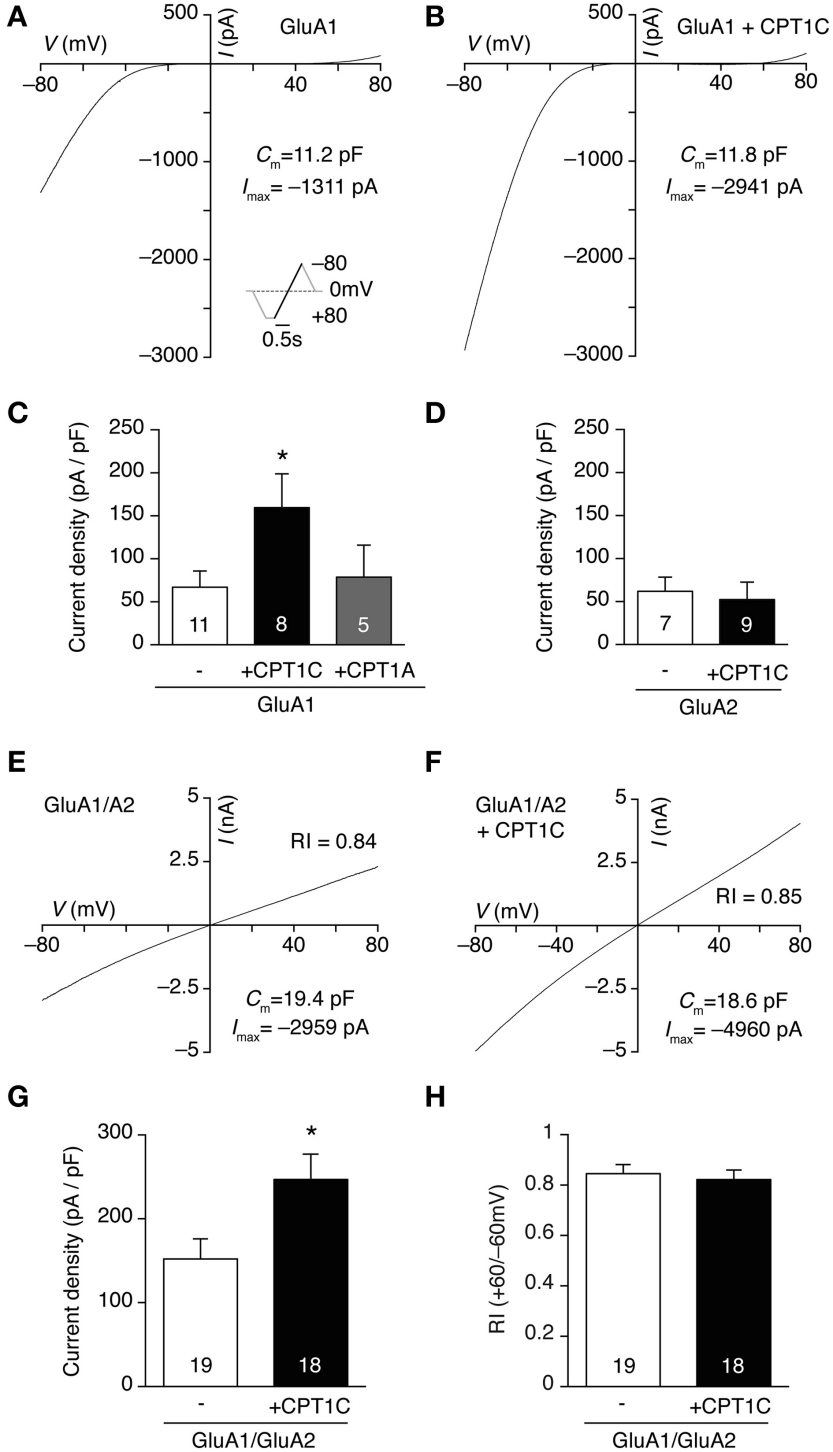
**CPT1C specifically increases glutamate-evoked currents of GluA1-containing AMPARs. (A)** Whole-cell current-voltage (IV) relationship for a tsA201 cell expressing GluA1 homomeric receptors. The IV plot was obtained by ramping membrane potential from −80 to +80 mV at a rate of 160 mV/ s in the presence of 1 mM glutamate plus 25 μM CTZ to avoid receptor desensitization. 100 μM spermine was added to the pipette solution. Inset represents the voltage protocol used. **(B)** Same as **(A)** but for a cell expressing GluA1 plus CPT1C-GFP (CPT1C). **(C)** Average normalized currents at -80 mV for GluA1 alone or together with CPT1C or CPT1A. GluA1 current density (-pA/pF) was increased by co-expression with CPT1C (^*^p < 0.05; Mann–Whitney *U*-test) but was unaffected by CPT1A co-expression (*p* > 0.05; Mann–Whitney *U*-test). Numbers in bars denote the number of recordings. **(D)** Average current density for GluA2 alone or with CPT1C (*p* > 0.05; Mann–Whitney *U*-test). Numbers in bars denote the number of recordings. **(E)** Whole-cell IV in the same conditions as **(A)** but for a cell expressing GluA1/GluA2 heteromeric AMPARs. Rectification index (RI; I_+60mV_/I_−60mV_) gives a read-out of GluA2 incorporation. **(F)**. Same as **(E)** but for a cell expressing GluA1/GluA2 plus CPT1C. **(G)**. Average normalized currents at −80 mV for GluA1/GluA2 AMPARs with or without CPT1C. GluA1/A2 current density (-pA/pF) was increased by co-expression with CPT1C (^*^p < 0.05; Mann–Whitney *U*-test). Numbers in bars denote the number of recordings. **(H)**. Average RI for the cells recorded in both conditions showing no differences between groups.

Previous results (Schwenk et al., 2012) and our coimmunoprecipitation experiments suggest that CPT1C and AMPARs form part of the same complex at some stage either during the biosynthetic pathway or as an integral part of the receptor at the cell surface. Given that, we wondered if the interaction between AMPAR subunits and CPT1C could have any functional consequences on AMPARs. To explore that possibility we transiently transfected tsA201 cells with GluA1 subunit in the absence and presence of CPT1C-GFP and we measured glutamate-evoked whole-cell currents at different holding potentials by applying a voltage ramp in the presence of 1 mM glutamate and 25 μM CTZ. Figures 2A,B illustrate two examples of currents recorded between −80 and +80 mV in cells transfected with GluA1 and GluA1+CPT1C, respectively. Our results show that whole-cell current density measured at −80 mV from cells expressing GluA1 together with CPT1C was higher than those currents recorded for GluA1 alone (Figure 2C; 66.97 ± 18.77 pA/pF for GluA1 vs. 159.4 ± 39.43 pA/pF for GluA1+CPT1C; *p* = 0.0431; Mann–Whitney *U*-test; *n* = 11 and 8 respectively).

For the coimmunoprecipitation experiments performed in Figure 1 we used a GFP-tagged form of CPT1C due to the lack of a good commercially available anti-CPT1C antibody. To be consistent between experiments we carried out the electrophysiological experiments with the GFP-tagged form of CPT1C. However, we could not rule out that the fused GFP could somehow account for the whole-cell current increase seen in our recordings. To exclude that possibility we repeated our experiments with a non-tagged form of CPT1C protein (CPT1C-pIRES; see methods). We found a similar increase in the current density to that obtained with the GFP-tagged form (data not shown). Further, the effect of CPT1C on GluA1 could be due to a general CPT1 activity or feature and not due to the specific interaction of the isoform CPT1C with GluA1. To test this possibility we overexpressed the most ubiquitous form of CPT1, CPT1A, together with GluA1. We found similar current density values to those obtained with GluA1 alone (Figure 2C; 66.97 ± 18.77 pA/pF for GluA1 vs. 78.69 ± 37.10 pA/pF for GluA1+CPT1A; *p* = 1.0000; Mann–Whitney *U*-test; *n* = 11 and 5 respectively). This demonstrates the specificity of the CPT1C effect on AMPAR-mediated currents.

CPT1C is also present in AMPAR complexes isolated with GluA2 antibody (Schwenk et al., 2012 and Figure 1B). Therefore we investigated whether the effect of CPT1C on GluA2 subunit was similar to the one seen for GluA1 subunits by doing whole-cell density current experiments in GluA2 homomeric AMPARs. Unlike GluA1, Figure 2D shows that there was no increase in current density when CPT1C was co-expressed with GluA2 in tsA201 cells (61.85 ± 16.48 pA/pF for GluA2 vs. 52.28 ± 20.27 pA/pF for GluA2+CPT1C; *p* = 0.2991; Mann–Whitney *U*-test; *n* = 7 and 9 respectively).

Most of glutamatergic neurons in CNS express GluA2-containing AMPARs either with GluA1, GluA3 or GluA4 (Gallo et al., 1992; Kondo et al., 1997; Lu et al., 2009; Reimers et al., 2011). Thus we decided to evaluate whether GluA1/GluA2 heteromeric receptors currents were similarly affected by CPT1C. Figures 2E–G shows that CPT1C was able to increase current density from heteromeric receptors (152.1 ± 23.94 pA/pF for GluA1/GluA2 vs. 246.9 ± 30.26 pA/pF for GluA1/GluA2+CPT1C; *p* = 0.0236; Mann–Whitney *U*-test; *n* = 19 and 18 respectively). In these experiments, in order to favor GluA2 presence in the receptor we transfected in a 1:2 ratio (GluA1:GluA2) and the GluA1 plasmid expressed the mCherry protein (see methods) thus allowing the recording of GluA1-containing receptors. In fact, for both conditions, the red fluorescence patched cells displayed linear responses, which were not different between both groups confirming the presence of GluA2 (Figure 2H; RI_+60/−60_ = 0.84 ± 0.04 without CPT1C vs. 0.82 ± 0.04 with CPT1C; *p* = 0.8910; Mann–Whitney *U*-test; *n* = 19 and 18 respectively).

### CPT1C does not alter gating properties of AMPARs

In the current density experiments described above we found a significant increase in the glutamate-evoked GluA1-mediated currents when CPT1C was present. The total amount of current carried by a given population of receptors depends on several factors, which include the single channel conductance, the kinetics, the open probability of the receptor and the number of receptors contributing to the current. Any alteration in these parameters might result in changes in the current magnitude. So, one possibility would be that CPT1C could modulate the single channel conductance or open probability of AMPARs either by a direct interaction (Soto et al., 2007, 2014; Suzuki et al., 2008; Coombs et al., 2012; Shelley et al., 2012) or indirectly by phosphorylation (Derkach et al., 1999; Banke et al., 2000; Kristensen et al., 2011). So, we decided to investigate the mechanisms contributing to the current increase observed in AMPARs together with CPT1C. To determine whether AMPAR single channel conductance was altered by CPT1C we transfected GluA1 either alone or together with CPT1C in tsA201 cells and applied fast applications of glutamate (10 mM; 100 ms duration; 1 kHz) onto out-side out patches followed by non-stationary fluctuation analysis of the glutamate-evoked responses.

**Figure 3:**
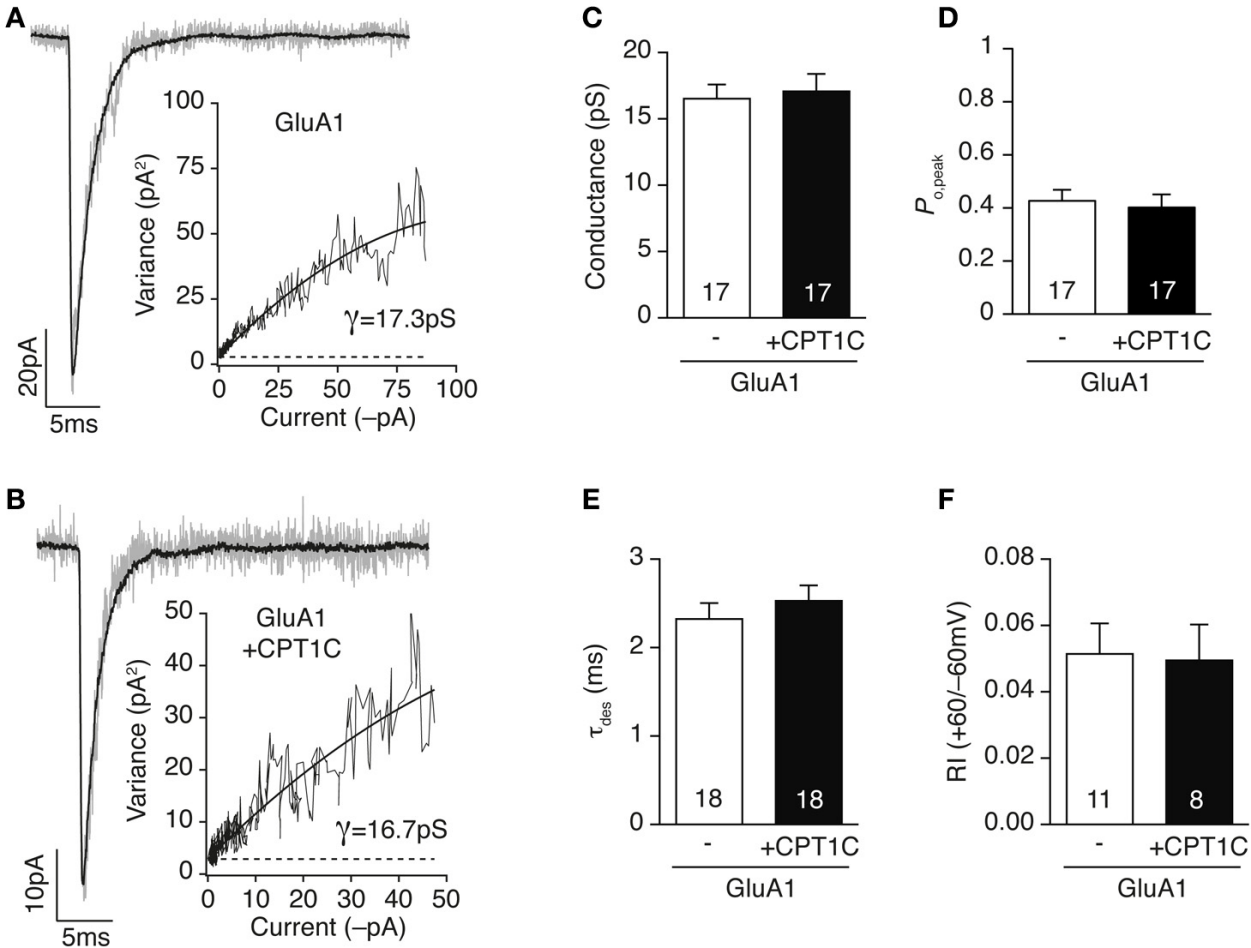
**CPT1C does not alter AMPAR gating properties. (A)** Current activated by rapid application of 10 mM glutamate (100 ms, −60 mV) to an outside-out patch from a tsA201 cell expressing GluA1 alone. Gray trace represents a single response and black line the average of 37 responses. Inset shows the current variance vs. mean current plot for this patch. The weighted mean single-channel conductance estimate for this record was 17.3 pS. **(B)** Same as **(A)** but for a cell expressing GluA1 plus CPT1C-GFP (CPT1C). Black line is the average of 38 responses. Inset: current variance vs. mean current plot for this patch giving a weighted mean single-channel conductance of 16.7 pS. **(C–F)** CPT1C has no influence on either weighted single channel conductance (**C**; pS), peak open probability (**D**; *P*_o,peak_), desensitization kinetics (**E**; τ_des_) or rectification index (**F**; I_+60mV_/I_−60mV_) of GluA1 homomeric AMPARs. *P* > 0.05, Mann–Whitney *U*-test for all groups. Numbers in bars denote the number of recordings.

Figures 3A,B show typical responses for GluA1 homomers alone (Figure 3A) or together with CPT1C (Figure 3B). The single channel conductance of GluA1 homomers was not altered when co-expressed with CPT1C (Figure 3C; 16.53 ± 1.07 pS for GluA1 alone vs. 17.07 ± 1.31 pS for GluA1+CPT1C; *p* = 0.8095, Mann–Whitney *U*-test; *n* = 17 for both). CPT1C did not alter peak open probability (*P*_o,peak_) of AMPARs (Figure 3D; 0.43 ± 0.04 for GluA1 vs. 0.40 ± 0.05 for GluA1+CPT1C; *p* = 0.6052, Mann–Whitney *U*-test; *n* = 17 for both). Similarly, the AMPARs kinetics measured as the desensitization decay time constant (see methods) were not changed (Figure 3E; 2.32 ± 0.18 ms for cells expressing GluA1 alone vs. 2.53 ± 0.17 ms for cells expressing GluA1+CPT1C; *p* = 0.3112, Mann–Whitney *U*-test; *n* = 18 for both). Since AMPAR auxiliary subunits attenuate the strong block by polyamine of calcium permeable AMPARs (CP-AMPARs) (Soto et al., 2007, 2009), we evaluated if CPT1C was able to have a similar effect. We therefore measured the rectification index (RI) at +60/–60 mV (see methods) from the IV ramps experiments (Figures 2A–C), however we did not see any alteration in the strong inwardly rectifying IV relationship of CP-AMPARs (Figure 3F; 0.051 ± 0.009 for cells expressing GluA1 alone vs. 0.049 ± 0.011 for cells expressing GluA1+CPT1C; *p* = 0.8554, Mann–Whitney *U*-test; *n* = 11 and 8 respectively).

Taken together, data from Figures 2, 3 show that glutamate-evoked current density is increased by co-expression of CPT1C in GluA1 expressing cells but not those expressing only GluA2 subunit. Nonetheless, GluA1 channel properties (single channel conductance, peak open probability and desensitization kinetics) are unaffected suggesting an increase in receptor number at the cell surface. These results could also be indicating that both proteins do not associate at the plasma membrane despite a larger amount of current when CPT1C is co-expressed with GluA1.

### AMPARs co-localize with CPT1C at the ER but not at the plasma membrane

**Figure 4:**
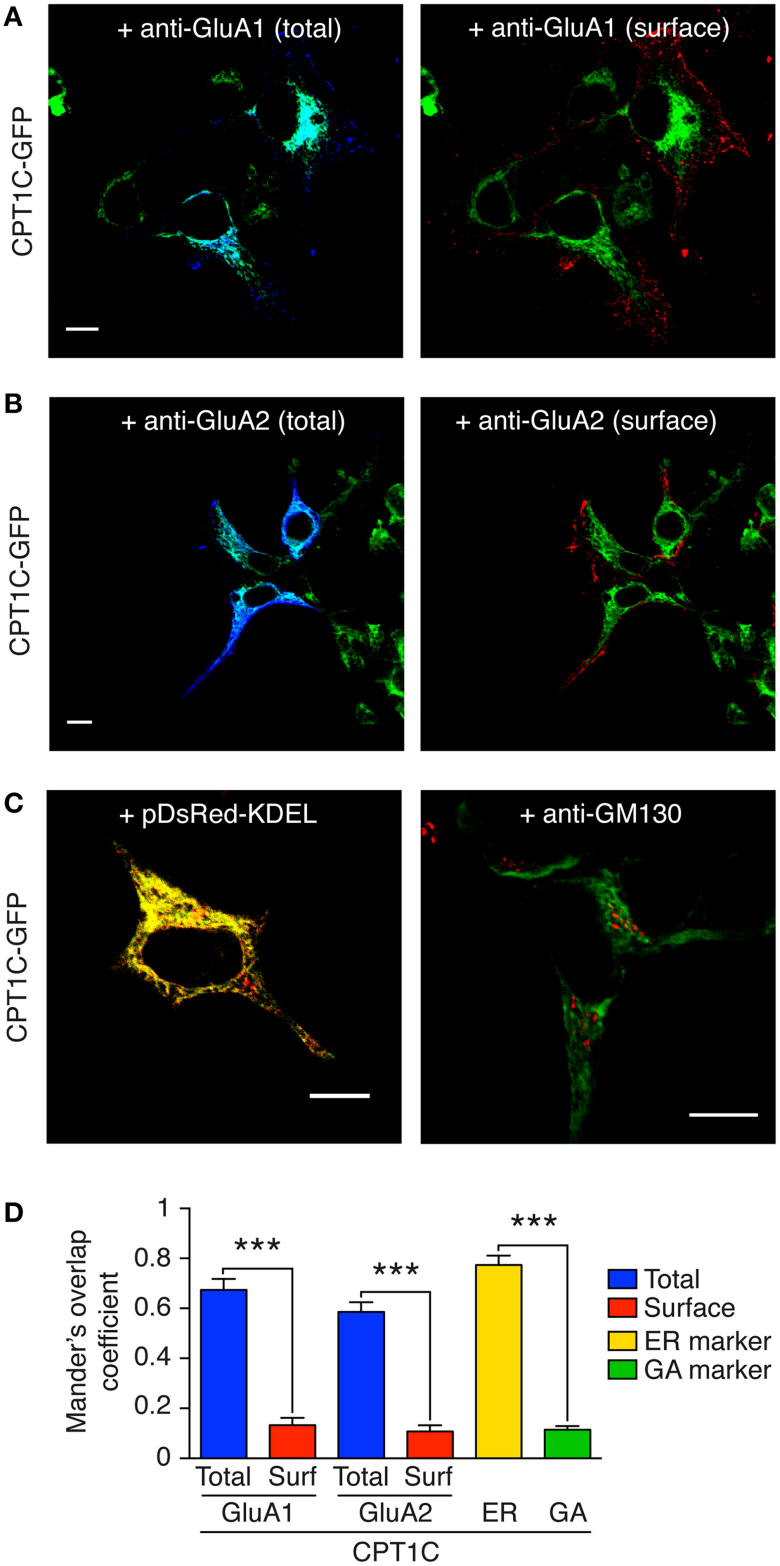
**GluA1 and GluA2 co-localize with CPT1C at intracellular compartments but not at the plasma membrane**. CPT1C does not co-localize with the Golgi Apparatus marker GM-130. **(A)** Confocal image showing co-localization of CPT1C-GFP (green) with GluA1 (dark blue signal in left panel) in transfected tsA201 cells. Co-localization signal is displayed as light blue (left panel). There is no co-localization between CPT1C (green) and cell-surface GluA1 (red signal in right panel). Scale bar: 10 μm. **(B)** Confocal image showing co-localization of CPT1C (green) with GluA2 (dark blue signal in left panel) in transfected tsA201 cells. Co-localization signal is apparent as light blue color (left panel). There is no co-localization between CPT1C (green) and cell-surface GluA2 (red signal in right panel). Scale bar: 10 μm. **(C)** Co-localization (yellow signal in left panel) of CPT1C-GFP with ER marker (pDsRed-KDEL). Lack of co-localization (absence of yellow signal in right panel) of CPT1C (green) with the Golgi Apparatus marker GM-130 (red) in transfected tsA201 cells. Scale bars: 10 μm. **(D)** Representation of co-localization values quantified by Manders Overlap Coefficient (MOC; see Methods) expressed as mean ± SEM. MOC values for total GluAs and CPT1C (blue columns) were statistically different from surface GluAs and CPT1C (red columns; ^***^p < 0.001 for both comparisons; Mann–Whitney *U*-test; *n* = 10 for all conditions). MOC values for CPT1C-ER marker (pDsRed-KDEL) show strong co-localization (yellow bar). MOC values for CPT1C-GM130 (GA marker; green bar) were similar to those of surface GluAs-CPT1C.

Our results show a functional effect of CPT1C on GluA1. This effect though, seems not to be similar to that of “bona fide” auxiliary subunits, which exert several modulatory effects on AMPAR at the plasma membrane level (Jackson and Nicoll, 2011; Shanks et al., 2012). Therefore, to study the presence or absence of CPT1C at the cell surface, we visualized the location of the interaction between CPT1C and AMPAR subunits. With this aim, we co-transfected cells with CPT1C-GFP and GluA1 or GluA2 and performed immunofluorescence to differentially visualize surface AMPARs (red in Figures 4A,B) and the total pool of AMPARs subunits (blue in Figures 4A,B) by using confocal microscopy (for details, see methods).

Results in Figures 4A,B using cell lines indicate that CPT1C co-localize with both intracellular GluA1 and GluA2 (Figures 4A,B; light blue in left panels). Quantification of these co-localizations using Manders Overlap coefficient (MOC; see methods) is shown in Figure 4D (0.67 ± 0.14 for GluA1 and 0.59 ± 0.12 for GluA2; blue columns; *n* = 10). In fact, CPT1C expression seems to be restricted to areas close to the nucleus and with a reticular pattern (Figures 4A,B; green signal). Interestingly, there is no co-localization of CPT1C with surface receptors confirming that the interaction does not take place at the cell-surface (Figures 4A,B; right panels). Figure 4D (red columns) shows MOC for CPT1C and surface GluA1 (0.13 ± 0.09; *n* = 10) or surface GluA2 (0.11 ± 0.08; *n* = 10).

CPT1C has been described to co-localize with ER markers in transfected cell lines and in neurons (Sierra et al., 2008; Carrasco et al., 2012). We confirmed this previous localization of CPT1C in the ER in our expression system (Figure 4C; left panel and Figure 4D; yellow column; MOC of 0.77 ± 0.04). Since GluA subunits dwell in the Golgi Apparatus during posttranscriptional modifications (Greger et al., 2002) we wondered if CPT1C and GluAs might interact at this level. However, we found that CPT1C shows poor co-localization with a Golgi Apparatus marker (GM-130) as shown in Figure 4C (right panel) and Figure 4D (green column; MOC of 0.11 ± 0.05; *n* = 10).

This data clearly demonstrates that GluA subunits are together with CPT1C at the ER level but not at the plasma membrane.

### Surface expression of GluA1 is increased in the presence of CPT1C

Our results showing that AMPAR gating at the plasma membrane level is not altered by CPT1C suggests that the increase in whole-cell currents might be explained by an increased number of receptors present at the cell surface.

**Figure 5:**
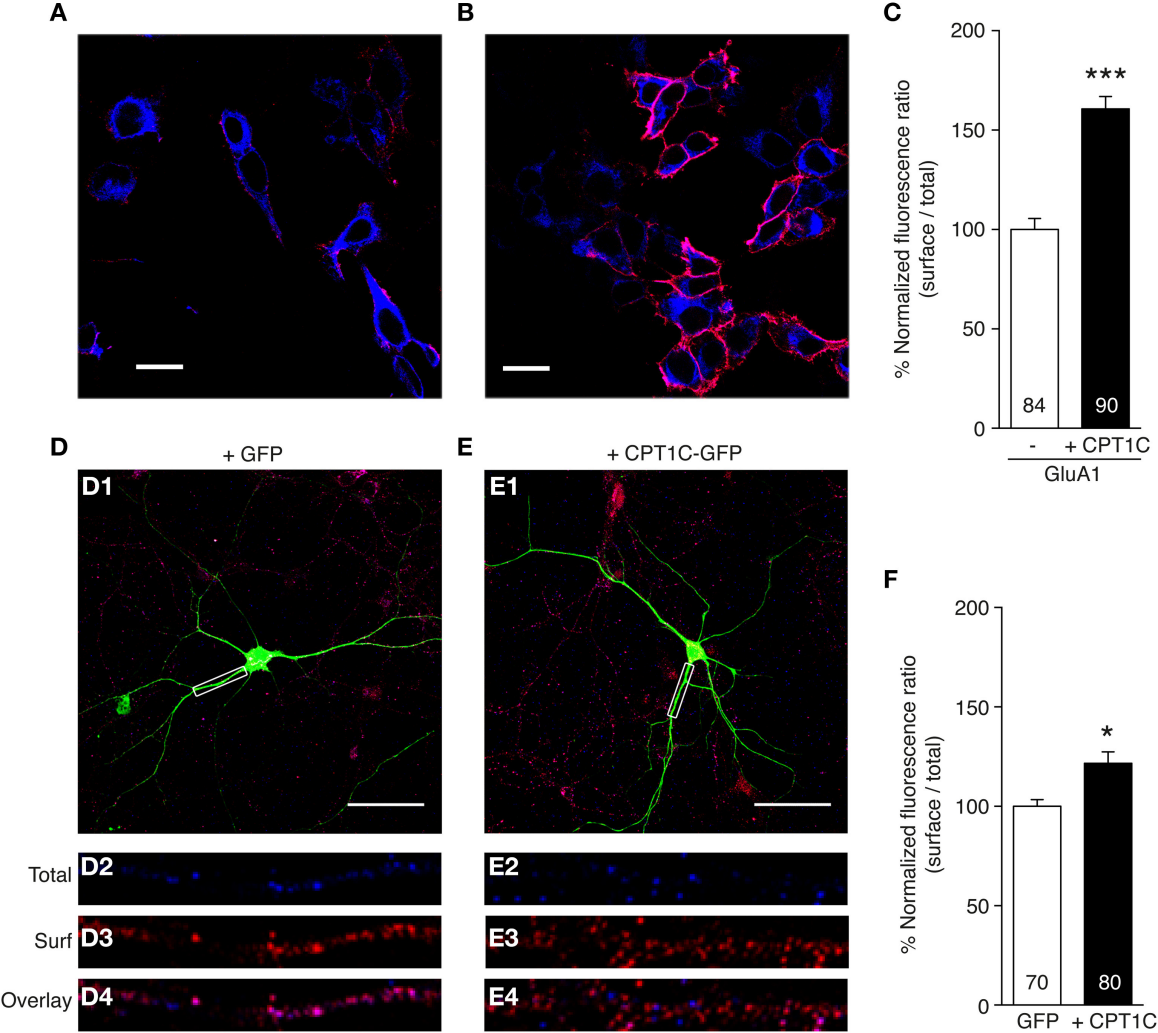
**CPT1C enhances surface expression of GluA1. (A,B)** Representative single confocal images of tsA201 cells co-expressing GluA1 and GFP **(A)** or GluA1 and CPT1C-GFP **(B)**. Surface GluA1 was labeled in live cells with anti-GluA1-NT and Alexafluor 555 (red signal in the images). Subsequently cells were permeabilized and total GluA1 expression level was labeled with the same primary antibody but with Alexafluor 647 (blue signal in the images). Scale bars: 20 μm. **(C)** Quantification of the GluA1 surface to total ratio normalized to GluA1, expressed as a percentage. GluA1 surface expression was increased by co-expression with CPT1C (^***^p < 0.001; Mann–Whitney *U*-test). Data are means ± SEM. Numbers in bars denote the number of cells quantified from 4 different immunofluorescence experiments. **(D)** Example of a DIV 10 cortical neuron transfected with GFP. Surface GluA1 was labeled in fixed cells with anti-GluA1-NT and Alexafluor 555 (red signal in the images). Subsequently cells were permeabilized and total GluA1 expression level was labeled with the same primary antibody but with Alexafluor 647 (blue signal in the images). D1: field image showing the GFP in green, the surface GluA1 in red and the total GluA1 in blue. Scale bar: 50 μm. D2: magnification of the dendritic boxed area in D1 for total GluA1. D3: same as D2 but for surface GluA1. D4: overlay of intracellular and surface GluA1 for box in D1. **(E)** Example of a DIV 10 cortical neuron transfected with CPT1C-GFP in the same conditions as D. E1: field image showing the CPT1C-GFP in green, the surface GluA1 in red and the total GluA1 in blue. Scale bar: 50 μm. E2: magnification of the dendritic boxed area in E1 for total GluA1. E3: same as E2 but for surface GluA1. E4: overlay of total and surface GluA1 for box in E1. **(F)** Quantification of endogenous GluA1 surface to total ratio normalized to GFP transfected neurons, expressed as a percentage. GluA1 surface to total ratio was increased by overexpression of CPT1C-GFP (^*^p = 0.0226; Mann–Whitney *U*-test). Data represent means ± SEM. Numbers in bars denote the number of dendrites quantified from 3 different cultures.

To test this hypothesis we determined GluA1 surface expression using immunofluorescence quantification. We immunostained surface GluA1 in live transfected tsA201 cells (Figures 5A,B; red signal). We then permeabilized the cells and stained the total GluA1 pool (Figures 5A,B; blue signal). Given the variability in expression levels we calculated the ratio of the surface expression of GluA1 vs. the total expression level of GluA1 for the same cell.

As shown in Figure 5C the normalized ratio surface to total GluA1 was increased in cells co-expressing GluA1 and CPT1C-GFP (100 ± 5.53% for cells expressing GluA1 alone vs. 160.6 ± 6.24% for cells expressing GluA1+CPT1C; *p* < 0.0001, Mann–Whitney *U*-test; *n* = 84 and 90 cells respectively from 4 immunocytochemistry experiments for each condition). This result indicates a possible role of CPT1C in increasing GluA1 trafficking to the cell surface.

We wanted to extend these findings by studying CPT1C influence on surface expression of native AMPARs from neuronal cultures. Hence, we carried out immunofluorescence experiments in primary cortical neurons cultures at 10 DIV (Figures 5D–F). By using an equivalent methodology performed with tsA201 cells, we measured the GluA1 surface to total ratio in dendrites from GFP transfected neurons (Figure 5D) compared with CPT1C-GFP overexpressed neurons (Figure 5E). Figures 5D2–D4, E2–E4 show examples of the analyzed dendrites. Neurons transfected with CPT1C increased the GluA1 surface to total ratio (100 ± 3.58 % for control GFP transfected neurons vs. 118.8 ± 5.65 % for CPT1C-GFP transfected neurons; *p* = 0.0226; *n* = 70 and 80 dendrites respectively from 3 different cultures each; Figure 5F).

These results show that CPT1C favors the trafficking of GluA1-containing AMPARs in both heterologous cells and neurons.

### CPT1C enhancing effect on surface expression and current density is mediated by GluA1 C585

Our data suggest an effect of CPT1C on the trafficking of GluA1. This effect could be performed by a chaperone-like activity of CPT1C or by some posttranslational modification mediated by this protein directly on GluA1. It has been demonstrated that the posttranslational modification palmitoylation affects AMPAR trafficking (Hayashi et al., 2005; Lin et al., 2009; Yang et al., 2009). This modification consists in the reversible introduction of a lipid palmitate in some specific cysteine residues present in proteins. Given that it has been described that CPT1C can bind palmitoyl-CoA (Sierra et al., 2008) we addressed the question whether the observed increase in surface expression of GluA1 could be mediated by changes in the palmitoylation state of GluA1 due to CPT1C.

To check this possibility we first obtained two mutant forms of GluA1 that cannot be palmitoylated at previously described palmitoylable cysteine residues 585 and 811 (Hayashi et al., 2005) by changing the cysteine for a serine (C585S or C811S; see methods). We also tested the double mutant form GluA1(C585,811S). Hence, we studied the effect of these mutations and their co-expression with CPT1C-GFP in cell lines firstly using the immunofluorescence quantification of surface receptors and also studying the current density.

**Figure 6:**
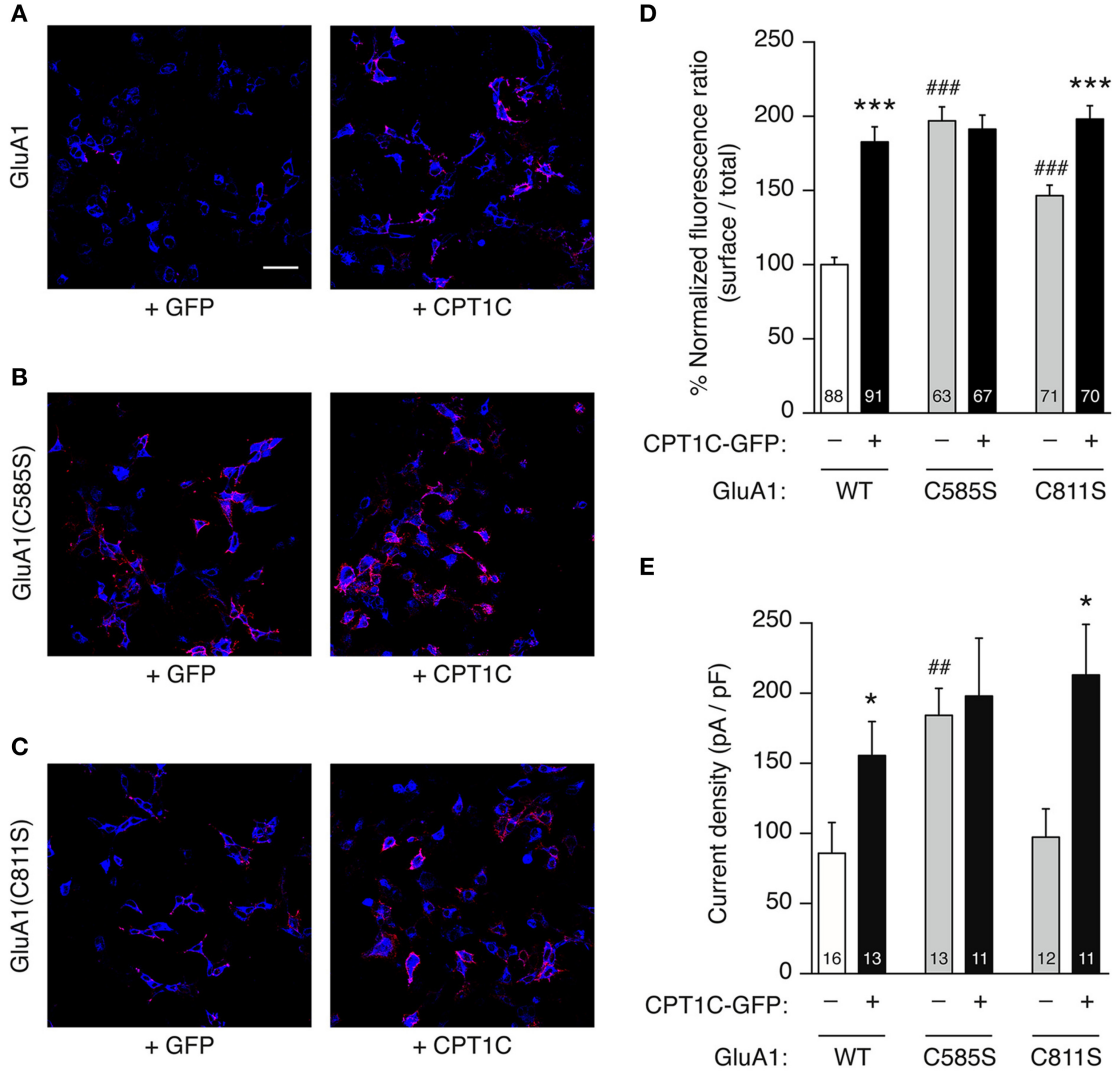
**GluA1 C585 is critical for the enhancement of current density and surface expression by CPT1C. (A–C)**. Representative single confocal images of tsA201 cells co-expressing different versions of GluA1 with (right panels) or without (left panels) CPT1C-GFP. In the images, surface GluA1 is shown in red and total GluA1 in blue. Scale bar: 50 μm. **(A)** Native GluA1 co-expressing GFP (+GFP) as the control condition, or CPT1C-GFP (+CPT1C). **(B)**. Same as **(A)** but for cells expressing GluA1 containing the point mutation C585S that abolishes palmitoylation at this residue. **(C)** Same as **(A,B)** but for GluA1 with the point mutation C811S. **(D)** Quantification of the GluA1 surface to total ratio normalized to GluA1, expressed as a percentage. GluA1 surface expression was increased by co-expression of CPT1C for both GluA1 and GluA1(C811S) (^***^p < 0.001; Mann–Whitney *U*-test) but not for GluA1(C585S). Non-palmitoylable forms of GluA1 increased surface expression of the receptors when compared to wild-type GluA1 (WT) (^###^*p* < 0.001; Mann–Whitney *U*-test). Numbers in bars denote the number of cells quantified from 3 different immunofluorescence experiments. **(E)** Averaged normalized currents at −80 mV for different versions of GluA1 alone or together with CPT1C. Current density (-pA/pF) was increased by co-expression of CPT1C with native GluA1 (WT) or mutant GluA1(C811S) (^*^p < 0.05; Mann–Whitney *U*-test) but not for GluA1(C585S). GluA1(C585S) increased the current density when compared to native GluA1 (^##^*p* < 0.01; Mann–Whitney *U*-test). Numbers in bars denote the number of recordings.

Figures 6A–C show confocal images of surface GluA1 quantification experiments for GluA1 constructs alone (left panels) and together with CPT1C (right images). Quantification of 3 immunocytochemistry experiments for each condition is presented in Figure 6D and Table 1. In parallel we carried out whole-cell current density experiments as described in Figure 2 to directly assess the effect of CPT1C on the glutamate-evoked currents of GluA1 mutants (Figure 6E and Table 2).

**Table 1:** **Normalized fluorescence ratio (% of surface vs. total) values**.

**Receptor**		**Normalized fluorescence ratio (Surface/Total) (%)**	***n***	***p*-value (vs. no CPT1C)**	***p*-value (vs. GluA1)**
GluA1	−	100.0 ± 4.86	88		
	+CPT1C	182.6 ± 10.25	91	<0.001	
GluA1(C585S)	−	196.9 ± 9.41	63		<0.001
	+CPT1C	191.3 ± 9.47	67	0.4876	
GluA1(C811S)	−	146.5 ± 7.08	71		<0.001
	+CPT1C	198.2 ± 8.99	70	<0.001	
GluA1(C585,811S)	−	208.2 ± 11.55	35		<0.001
	+CPT1C	208.1 ± 7.87	37	0.9014	

Transiently transfected tsA201 cells for different conditions were immunostained against surface and total GluA1 (see methods) and the ratio of GluA1 surface expression vs. GluA1 total expression was calculated and normalized to that of GluA1 control group. Three different immunofluorescence experiments were performed for each condition and the total number of analyzed cells is shown (n). Each p value is from a Mann–Whitney U-test comparing normalized fluorescence values of the distinct GluA1 receptors in the absence or presence of CPT1C (“vs. no CPT1C” column) or normalized fluorescence values of mutant versions of GluA1 against wild type GluA1 (“vs. GluA1” column).

**Table 2:** **Current density values for GluA1 mutants with or without CPT1C**.

**Receptor**		**Current density (pA/pF)**	***n***	***p*-value (vs. no CPT1C)**	***p*-value (vs. GluA1)**
GluA1	−	85.86 ± 21.95	16		
	+CPT1C	155.5 ± 24.22	13	0.0334	
GluA1(C585S)	−	184.3 ± 19.19	13		0.0041
	+CPT1C	198.0 ± 41.25	11	0.7721	
GluA1(C811S)	−	97.32 ± 20.26	12		0.4166
	+CPT1C	213.0 ± 36.12	11	0.0210	
GluA1(C585,811S)	−	153.8 ± 14.58	10		0.0143
	+CPT1C	178.9 ± 34.89	10	0.8534	

Transiently transfected tsA201 cells for different conditions were whole-cell patch clamped and voltage ramps from −80 to +80 mV were applied (see methods for details). The maximum current at −80 mV was then normalized against the cell capacitance to obtain the current density values (-pA/pF). The total number of recorded cells is shown (n). Each p value is from a Mann–Whitney U-test comparing current density values for each GluA1 receptor in the absence or presence of CPT1C (“vs. no CPT1C” column) or current density values of mutant versions of GluA1 against wild type GluA1 (“vs. GluA1” column).

We replicated the immunofluorescence and electrophysiology results obtained in Figures 2, 5 in parallel with the mutant forms of GluA1. As previously found, CPT1C increased the surface/total ratio of GluA1 (100.0 ± 4.86% for GluA1 vs. 182.6 ± 10.25% for GluA1+CPT1C; *p* < 0.001; Figure 6D and Table 1) and the current density of homomeric GluA1 (85.86 ± 21.95 pA/pF for GluA1 vs. 155.5 ± 24.22 pA/pF for GluA1+CPT1C; *p* = 0.0334; Figure 6E and Table 2).

We observed that in the absence of CPT1C, GluA1(C585S) expression was enhanced at the cell surface by 1.97-fold compared to native GluA1 (*p* < 0.001; Figure 6D and Table 1) and that the glutamate-evoked current carried by GluA1(C585S) was increased to the same degree (2.15-fold; *p* = 0.0041, Figure 6E and Table 2). These results are in keeping with previous findings (Hayashi et al., 2005). Interestingly, the effects of CPT1C co-expression and the GluA1(C585S) mutation were not additive. Specifically, CPT1C did not further increase the surface expression of GluA1(C585S) (*p* = 0.4876, Figure 6D and Table 1) or the current density of GluA1(C585S) (*p* = 0.7721, Figure 5E and Table 2). Likewise CPT1C did not vary the high surface expression or further enhance current density of the double mutant GluA1(C585,811S) (*p* = 0.9014 and *p* = 0.8534, Tables 1, 2). This suggests that C585 might be crucial in the CPT1C effect on trafficking of GluA1.

In addition, GluA1(C811S) surface expression was also enhanced compared to GluA1 (*p* < 0.0001; Figure 6D and Table 1). Nevertheless, although significantly different from GluA1, GluA1(C811S) seems to be less efficiently expressed at the membrane surface compared the other GluA1 mutants (*p* < 0.001 for GluA1(C811S) vs. GluA1(C585S) or GluA1(C585,811S); Mann–Whitney *U*-test for both comparisons) and clearly GluA1(C811S) was not able to increase current density compared with GluA1 (*p* = 0.4166, Figure 6E and Table 2). However CPT1C co-expression does have an effect on both GluA1(C811S) surface expression (146.5 ± 7.08 % for GluA1(C811S) vs. 198.2 ± 8.99 % for GluA1(C811S)+CPT1C; *p* < 0.001; Figure 6D and Table 1) and current density (97.32 ± 20.26 pA/pF for GluA1(C811S) vs. 213.0 ± 36.12 pA/pF for GluA1(C811S)+CPT1C; *p* = 0.0210; Figure 6E and Table 2).

Finally, the effect of CPT1C on GluA1(C811S) is equivalent to the effect of the C585S mutation alone in terms of both, surface expression (Figure 6D; *p* = 0.9623) and current density (Figure 6E; *p* = 0.6430). These results suggest that the enhancing effect of CPT1C on surface expression and current density is mediated by a modification of cysteine 585 of GluA1 subunits.

### Palmitoylation state of GluA1 is unaffected by CPT1C overexpression

It has been previously demonstrated that palmitoylation of GluA1 at the C585 residue retains AMPARs in the Golgi Apparatus (Hayashi et al., 2005) implying that depalmitoylated GluA1 at C585 traffics more efficiently to the plasma membrane. Our data corroborate these findings since the number of receptors at the surface in the mutant C585S (where cysteine 585 cannot be palmitoylated) is increased. Interestingly, the surface expression of GluA1(C585S) is approximately the same as GluA1(C811S) expressed with CPT1C (where CPT1C effect can only be on the intact cysteine 585). This result seems to point to CPT1C being a potential depalmitoylating enzyme of GluA1.

**Figure 7:**
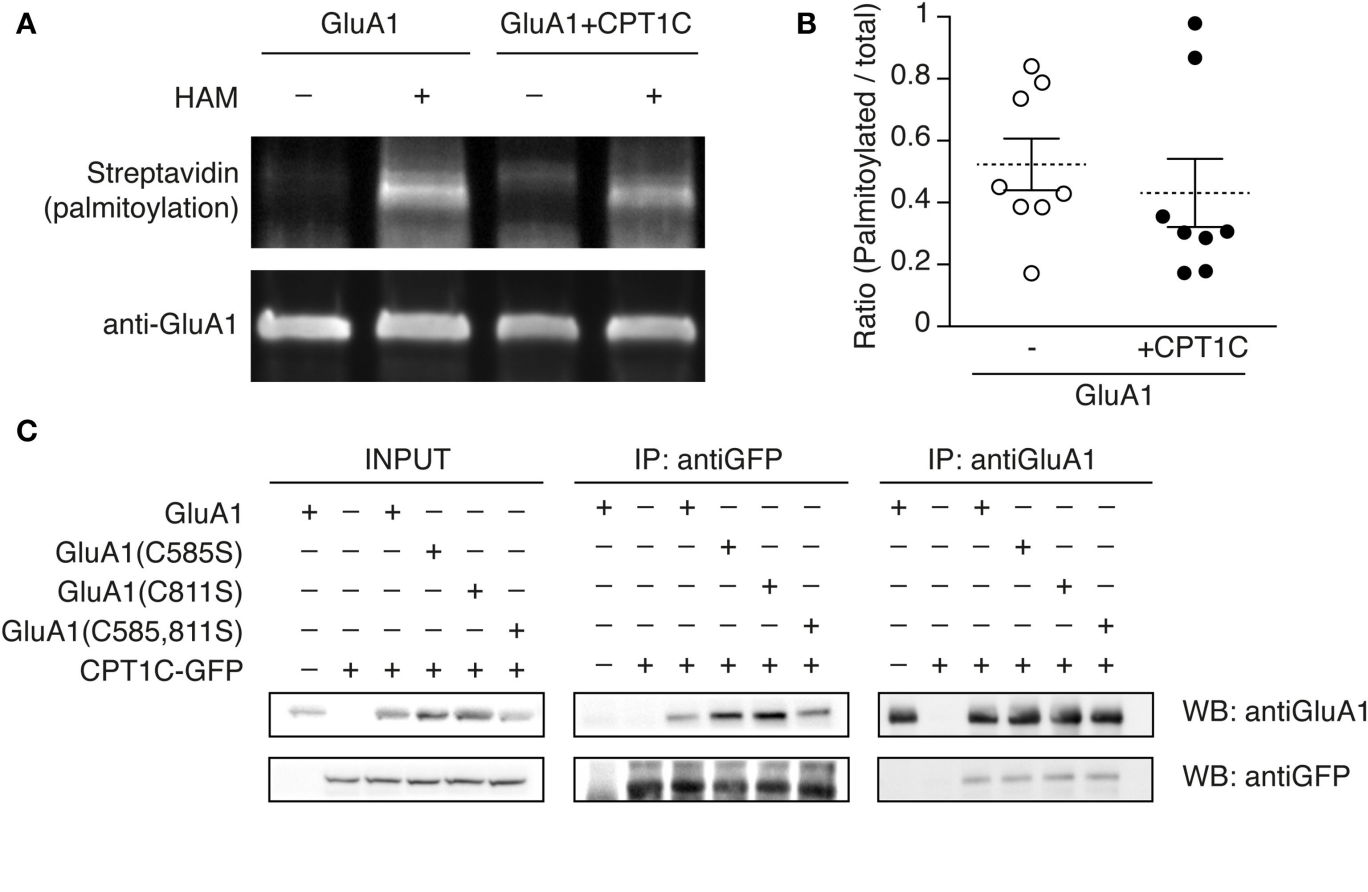
**GluA1 palmitoylation state is not altered by CPT1C and does not affect the interaction with GluA1. (A)** Palmitoylation levels of GluA1 alone (GluA1) and together with CPT1C-GFP (GluA1+CPT1C), in transfected tsA201 cells, detected by means of Acyl-Biotin Exchange (ABE). The thiol-biotinylated immunoprecipitates of GluA1 following the ABE assay for both transfected conditions were subjected to SDS-PAGE. Palmitoylation of GluA1 can only be detected in plus-hydroxilamine (+HAM) samples. Minus -HAM samples control non-specific incorporation of biotin. GluA1 palmitoylation levels (top) were detected by Western blotting with streptavidin-HRP (palmitoylation). After stripping the membranes the total amount of immunoprecipitated GluA1 was detected by Western blotting with anti-GluA1-NT antibody (anti-GluA1, bottom). **(B)** Quantification of palmitoylation levels for GluA1 alone (open circles) or GluA1 plus CPT1C (filled circles) in tsA201 cells. Ratio of palmitoylated GluA1 to total GluA1 for each single experiment is shown together with mean (discontinuous horizontal lines) and SEM (continuous vertical lines) (*p* > 0.05; Mann–Whitney *U*-test; *n* = 8 for both). **(C)** Co-IP of the membranous fraction of tsA201 cells co-expressing GluA1 wild type or non-palmitoylable mutants—GluA1(C585S), GluA1(C811S), and GluA1(C585,811S)—together with CPT1C-GFP. The interaction between CPT1C and GluA1 is not dependent on palmitoylation of C585 or C811 residues. As negative controls GluA1 was co-expressed with an empty plasmid expressing GFP alone (first lanes of the boxes) and CPT1C-GFP was co-expressed with an empty pDsRed (second lanes from the boxes). Transfected cells were lysed and membranes were solubilized as described in Figure 1 and methods. An input sample collected prior to immunoprecipitation of these extracts is shown as “INPUT.” Inputs and immunoprecipitated samples were separated and Western Blotted as described in Figure 1. Immunoprecipitations were replicated three times.

To test this hypothesis we analyzed the palmitoylation level of GluA1 when expressed in the absence or presence of CPT1C. We performed the Acyl Biotin Exchange assay as described in Brigidi and Bamji (2013) (see methods). This assay allows the replacement of a pre-existing palmitate bound to cysteines of a given protein with a biotin group. The biotin is subsequently detected with streptavidin to give a read-out of palmitoylation levels. Therefore we transfected tsA201 cells with GluA1 alone or GluA1 plus CPT1C-GFP. Palmitoylation levels of GluA1 in the absence of CPT1C were equivalent to GluA1 palmitoylation levels in the presence of CPT1C (Figure 7A; upper panel, second and forth lane and Figure 7B). Immunoprecipitated GluA1 was quantified to normalize palmitoylation levels (Figure 7A; lower panel). Figure 7B display the single experiments ratio values of palmitoylated GluA1 vs. total GluA1 for both conditions where it can be observed there is no significant difference (0.523 ± 0.084 for GluA1 vs. 0.431 ± 0.11 for GluA1+CPT1C; *p* = 0.3228; *n* = 8 for both).

Thus, by using this methodology we could not confirm the role of CPT1C as a depalmitoylating enzyme.

Given that C585 seems critical to favor AMPARs trafficking but its palmitoylation state is not changed by CPT1C we wondered if the lack of palmitate group could perhaps interfere with the physical interaction between GluA1 and CPT1C. In order to check if CPT1C ability to interact with GluA1 was eliminated when residue 585 was non-palmitoylated, we did co-IP assays with the GluA1(C585S) and CPT1C. As shown in Figure 7C, GluA1 and CPT1C retained the ability to interact even when cysteines 585, 811 or both were mutated to non-palmitoylable forms. This result indicates that the binding of both proteins depends on other domains/residues and that palmitoylable C585 does not determine this interaction despite its importance in CPT1C effect on trafficking properties of AMPARs.

## Discussion

In this study we describe a novel function of CPT1C in regulating AMPAR surface expression in both heterologous cells and neurons. In tsA201 cells CPT1C increases whole-cell glutamate-evoked currents of homomeric GluA1 and heteromeric GluA1/GluA2 AMPARs. Moreover, CPT1C overexpression enhances the number of endogenous AMPARs trafficked to the dendritic surface in rat cortical neurons. This trafficking effect is specific to the brain isoform CPT1C since the canonical CPT1 isoform also expressed in neurons (CPT1A) is not able to increase GluA1 mediated currents. Additionally, CPT1C modulation seems to be subunit specific since GluA2 homomeric AMPARs are unaffected by CPT1C co-expression. Despite GluA1 and CPT1C coimmunoprecipitating, both proteins do not co-localize at the plasma membrane level and no further biophysical modulation of AMPARs by CPT1C exists. Finally, the palmitoylable cysteine 585 of the GluA1 subunit seems to be crucial for the CPT1C alteration of AMPARs trafficking properties although no changes in the palmitoylation state of the receptor seem to occur.

### Role of CPT1C in AMPARs trafficking

In our experiments, both current density and cell surface expression are increased when CPT1C is co-expressed with GluA1. Despite this, GluA2 homomeric AMPARs seem not to be regulated by CPT1C, while GluA2-containing AMPARs, which are the most abundant form of AMPARs in neurons, are also sensitive to CPT1C. This points to a significant role of CPT1C in the delivery of GluA1 to the membrane in neurons. Indeed, the important role of CPT1C in synaptic transmission is evident since CPT1C knock-out mice have spatial learning problems, motor impairment and hypoactivity (Carrasco et al., 2012, 2013). Alterations in AMPAR-mediated signaling might underlie these phenotypes since in immature spines AMPAR content is low compared with mature synapses (Petralia et al., 1999). In fact, CPT1C KO animals show poor dendritic spine maturation in hippocampal neurons (Carrasco et al., 2012). In agreement with low AMPAR content at synapse level, recent unpublished electrophysiological data by our group has proved that synaptic transmission is altered in CPT1C KO animals (*submitted manuscript*). In these animals the lack of CPT1C translates to less efficient synaptic trafficking since AMPAR-mediated mEPSCs in pyramidal hippocampal neurons are diminished. All these findings reveal an important relationship between CPT1C and AMPARs. Our data and the functional evidence from the KO studies together with the ability of CPT1C to interact with AMPARs (Figure 1 and Schwenk et al., 2012) makes CPT1C a suitable candidate to be a regulating partner of AMPARs and an important protein for the correct function of AMPARs.

### Subunit specificity of CPT1C modulation

Our coimmunoprecipitation experiments showed direct interaction of CPT1C with both GluA1 and GluA2 subunits. Nevertheless the observed effects of CPT1C on whole-cell currents and surface expression were specific for GluA1-containing AMPARs since the results were not replicated in cells expressing GluA2 homomeric AMPARs. GluA1 and GluA2 subunits have distinct features in their structure including the Q/R site and the intracellular C-terminal domain, which translate to important functional differences (Traynelis et al., 2010). Remarkably, the important C585 palmitoylable residue in GluA1 (C610 in GluA2) for CPT1C effect is located +3 aminoacids from the crucial Q/R site. Another significant difference is the short C-terminal domain of GluA2. In fact, variations in the C-tails and the Q/R site between both isoforms determine different trafficking properties of GluA1 and GluA2 (Greger et al., 2002; Henley and Wilkinson, 2013). Therefore, it could be possible that a differential modulation by CPT1C was dependent on the C-tail length, the specific C-terminal aminoacid composition or on Q/R site editing state of AMPARs. The majority of AMPARs in the CNS are heteromeric combinations (Lu et al., 2009; Traynelis et al., 2010) and trafficking properties are determined by the dominant effect of long forms of AMPARs (Henley and Wilkinson, 2013). Our results are in line with this dominant effect of long forms. However, it remains to be studied whether other features of GluAs account for the subunit selectivity. Future experiments with other AMPAR forms might unravel the subunit features accounting for the specific modulation of CPT1C. Moreover, it would be interesting to investigate whether CPT1C protein similarly modulates AMPARs together with auxiliary subunits.

It is noteworthy that the effect of CPT1C on GluA2-containing heteromeric AMPARs and native AMPARs—mostly heteromeric combinations containing GluA2—is less pronounced than the one observed for GluA1 homomeric AMPARs. Perhaps the 2-fold enhancement of GluA1 density at the cell membrane could be partially occluded in GluA2-containing receptors due to generally better trafficking properties of heteromeric combinations. Indeed, the current density values we obtained for heteromeric receptors were higher than for homomeric receptors (either GluA1 or GluA2). This reflects the fact that heteromeric combinations of AMPARs are favored at the expense of homomeric receptors when both subunits are present during the synthesis process at the ER (Cull-Candy et al., 2006). The enhanced trafficking of heteromeric combinations might translate into a less evident CPT1C influence on GluA2-containing receptors. Alternatively, stoichiometry might be an important determinant in CPT1C effect. This possibility could be studied in the future.

### CPT1C is not a genuine auxiliary subunit of AMPARs

From our results, it looks like that this new AMPAR interactor has a putative role in the delivery of AMPAR subunits to the cell surface. It has been described that many other AMPAR interacting proteins control AMPAR trafficking (Palmer et al., 2005; Anggono and Huganir, 2012; Lu and Roche, 2012). This is the case for auxiliary AMPAR subunits such as the TARPs that affect the channel properties of AMPARs while also playing an important role in surface trafficking (Nicoll et al., 2006). CNIH proteins also increase AMPARs surface expression (Schwenk et al., 2009) and modify the behavior of AMPARs both in expression systems and in neurons (Kato et al., 2010b; Coombs et al., 2012). Conversely this is not the case for CPT1C, as we have shown that this protein does not alter GluA1 channel properties. This fact is supported by the confocal imaging experiments where we could not see any co-localization between CPT1C and surface GluA1. Consequently CPT1C cannot be considered a TARP-like “bona fide” auxiliary subunit and it seems that its role is restricted to controlling AMPARs trafficking.

### CPT1C and AMPARs interact at the ER level

Even though CPT1C does not associate with AMPAR subunits at the plasma membrane level, it is clear that both proteins interact at some stage of the AMPAR synthesis pathway (Schwenk et al., 2012 and Figures 1, 4). The fact that CPT1C shows a clear ER pattern (Figures 4C–D; Sierra et al., 2008; Carrasco et al., 2012) makes this organelle a meeting point for both proteins where CPT1C could posttranslationally modify AMPARs accounting for the increased traffic to plasma membrane. Further, our co-localization studies also demonstrate that CPT1C does not seem to interact with GluA1 outside of the ER at all, as CPT1C does not co-localize with the Golgi Apparatus marker GM-130. Thus, our results suggest that the effect of the interaction of CPT1C/GluA1 might take place exclusively at the ER level. They also point out that the complex CPT1C-AMPAR would dissociate at some stage before AMPARs subunits move forward to the Golgi during their biosynthesis.

### Mechanisms underlying CPT1C modulation of AMPARs: role of cysteine 585 of GluA1

AMPAR subunits are subject to several posttranslational modifications during biosynthesis that affect the trafficking of the receptors to the cell surface. This is the case for the reversible palmitoylation of AMPARs. All four AMPA receptor subunits are palmitoylated at two conserved sites, (C585 and C811 in GluA1) and palmitoylation/depalmitoylation of these two residues determine AMPARs trafficking properties (Hayashi et al., 2005; Lin et al., 2009; Yang et al., 2009). Given that CPT1s have palmitoylCoA as a substrate it seemed plausible to consider whether CPT1C was involved in a modification such as protein palmitoylation/depalmitoylation, thus potentially affecting AMPARs surface expression.

When studying the role of cysteine residues in CPT1C effect, we found that C585S mutation alone increased whole-cell currents and surface expression of GluA1 by 2-fold. These results are in accordance with previous ones demonstrating that depalmitoylation of AMPARs at C585 acts as a triggering signal for receptor forward trafficking (Hayashi et al., 2005). Interestingly, in the presence of CPT1C, the GluA1(C585S) no longer increased receptor trafficking. This points toward a crucial role of C585 residue for the CPT1C effect. This is confirmed by the fact that the GluA1(C811S) mutant is modulated by CPT1C to the same degree as GluA1 ruling out the involvement of C811 residue. Moreover the fact that CPT1C increases GluA1(C811S) trafficking to the same extent as GluA1(C585S) alone or with CPT1C suggests that the effect of CPT1C is dependent on the C585 residue.

Finally, our findings show that GluA1(C811S) increases surface expression when detected by immunofluorescence but current density is not increased in the same extent. This might be explained due to a different number of cells analyzed with each technique. Despite that discrepancy, the significant increment in both parameters when CPT1C is together with GluA1(C811S) indicates that GluA1 C811 is not crucial for the CPT1C effect.

### CPT1C does not alter GluA1 palmitoylation state

Given that CPT1C produces an increase in GluA1 surface expression to the same extent as the non-palmitoylable form of GluA1(C585S), we hypothesized that the effect of CPT1C on GluA1 subunits could be via depalmitoylation of C585. However, the palmitoylation state of GluA1 seems to be unaffected by co-expression with CPT1C, at least when detecting palmitoylation levels with the ABE assay. A possible issue with this methodology might be that the ABE assay not only detects palmitoylation but also other S-acylation modifications of GluA1, which have not yet been described. Therefore other techniques might be necessary to detect the palmitoylation levels of GluA1 C585 unambiguously. Alternatively, a necessary depalmitoylation process performed in the ER by CPT1C could be counteracted by additional palmitoylation of the receptor at other cell locations, thus making it difficult to detect changes in the palmitoylation state. Therefore, this hypothesis should be closely examined with future specific refinements of palmitoylation assays.

### How can CPT1C modulate AMPARs trafficking?

Though it appears that CPT1C does not depalmitoylate GluA1, the involvement of cysteine 585 is clear. It is possible therefore that the role of C585 is not related to the palmitoylation capacity of the amino acid. Perhaps the physical interaction of CPT1C with GluA1 masks the palmitate on this residue (or produces a conformational change in GluA1) facilitating its exit from the ER and the forward movement toward the Golgi. If that was the case, the masking would not be due to a direct interaction of CPT1C with the palmitate of C585 from GluA1, as both proteins still interact in the absence of this palmitate group. Otherwise, it may be possible that CPT1C acts as a chaperone during the synthesis of GluA1 and this chaperone effect could be related to the ER palmitoylation of C585. It has been described for other proteins (for instance the yeast polytopic membrane protein chitin synthase—Chs3) that the parallel action of palmitoyl acyl thioesterases and chaperones is necessary to achieve the correct folding and export from the ER (Lam et al., 2006) suggesting a relation between palmitoylation state and chaperone activity.

To gain insight into CPT1C modulation of AMPAR it would be important to elucidate the domains participating in the interaction. The topology of CPT1A shows that N- and C-terminal domains face the cytoplasm (Fraser et al., 1997). Presumably CPT1C displays the same topology thus restricting the interaction with AMPAR subunits to their C-terminal tail or transmembrane domains.

### CPT1C and disease

Even though CPT1C expression is restricted to the brain in healthy individuals, it functions as a stress-responsive gene under a variety of conditions, as its mRNA is up-regulated in cell lines from several different tissues as well as in mice following exposure to any one of a number of p53-activating stresses (Reilly and Mak, 2012). Therefore, it is suggested that CPT1C promotes cancer cell survival and tumor growth and it has been proposed as a new therapeutic target in cancer treatment. It is noteworthy that recent studies report an aberrant expression of CPT1C in gliomas (Cirillo et al., 2014; Wakamiya et al., 2014). These findings highlight the importance of unraveling the molecular mechanisms of CPT1C, which could be of great interest across a range of fields, for example in the study of new anticancer therapies and new diagnostic or prognostic markers.

## Author contributions

Esther Gratacòs-Batlle and David Soto designed the work; Esther Gratacòs-Batlle, Natalia Yefimenko, Helena Cascos-García and David Soto performed the research and analyzed the data; Esther Gratacòs-Batlle and David Soto interpreted the data and wrote the paper. This work is supported by: the Spanish Ministry of Science and Technology co-funded with European Union funds FEDER (grant BFU2011-24725), the European Commission (FP7-PEOPLE-2011-CIG; grant 293498) and the local Government of Generalitat de Catalunya (grant SGR 2009-152). David Soto is supported by the “Ramón y Cajal” Programme (RyC-2010-05970). We thank all members of the “Unitat d'Histologia,” especially the Head of the unit Professor Carles Solsona and Professor Joan Blasi for helpful assistance and discussion. We thank Dr. José Luis Rosa (Universitat de Barcelona) for the GM-130 antibody, Dr. Xavier Altajaf and Cristina Grau (IDIEBLL) for cortical neuronal cultures, Dr. Benjamín Torrejón (CCiTUB) for technical assistance with confocal microscopy, Dr. Francisco Ciruela for assistance with coimmunoprecipitation and Dr. Ian Coombs (University College London) for help and discussion.

### Conflict of interest statement

The authors declare that the research was conducted in the absence of any commercial or financial relationships that could be construed as a potential conflict of interest.
